# Clinical complexity and impact of the ABC (Atrial fibrillation Better Care) pathway in patients with atrial fibrillation: a report from the ESC-EHRA EURObservational Research Programme in AF General Long-Term Registry

**DOI:** 10.1186/s12916-022-02526-7

**Published:** 2022-09-02

**Authors:** Giulio Francesco Romiti, Marco Proietti, Marco Vitolo, Niccolò Bonini, Ameenathul Mazaya Fawzy, Wern Yew Ding, Laurent Fauchier, Francisco Marin, Michael Nabauer, Gheorghe Andrei Dan, Tatjana S. Potpara, Giuseppe Boriani, Gregory Y. H. Lip, on behalf of the ESC-EHRA EORP-AF General Long-Term Registry Investigators, L. Tavazzi, A. P. Maggioni, Z. Kalarus, R. Ferrari, A. Shantsila, A. Goda, G. Mairesse, T. Shalganov, L. Antoniades, M. Taborsky, S. Riahi, P. Muda, I. García Bolao, O. Piot, K. Etsadashvili, E. N. Simantirakis, M. Haim, A. Azhari, J. Najafian, M. Santini, E. Mirrakhimov, K. Kulzida, A. Erglis, L. Poposka, M. R. Burg, H. Crijns, Ö. Erküner, D. Atar, R. Lenarczyk, M. Martins Oliveira, D. Shah, E. Serdechnaya, E. Diker, D. Lane, E. Zëra, U. Ekmekçiu, V. Paparisto, M. Tase, H. Gjergo, J. Dragoti, A. Goda, M. Ciutea, N. Ahadi, Z. el Husseini, M. Raepers, J. Leroy, P. Haushan, A. Jourdan, C. Lepiece, L. Desteghe, J. Vijgen, P. Koopman, G. Van Genechten, H. Heidbuchel, T. Boussy, M. De Coninck, H. Van Eeckhoutte, N. Bouckaert, A. Friart, J. Boreux, C. Arend, P. Evrard, L. Stefan, E. Hoffer, J. Herzet, M. Massoz, C. Celentano, M. Sprynger, L. Pierard, P. Melon, B. Van Hauwaert, C. Kuppens, D. Faes, D. Van Lier, A. Van Dorpe, A. Gerardy, O. Deceuninck, O. Xhaet, F. Dormal, E. Ballant, D. Blommaert, D. Yakova, M. Hristov, T. Yncheva, N. Stancheva, S. Tisheva, M. Tokmakova, F. Nikolov, D. Gencheva, T. Shalganov, B. Kunev, M. Stoyanov, D. Marchov, V. Gelev, V. Traykov, A. Kisheva, H. Tsvyatkov, R. Shtereva, S. Bakalska-Georgieva, S. Slavcheva, Y. Yotov, M. Kubíčková, A. Marni Joensen, A. Gammelmark, L. Hvilsted Rasmussen, P. Dinesen, S. Riahi, S. Krogh Venø, B. Sorensen, A. Korsgaard, K. Andersen, C. Fragtrup Hellum, A. Svenningsen, O. Nyvad, P. Wiggers, O. May, A. Aarup, B. Graversen, L. Jensen, M. Andersen, M. Svejgaard, S. Vester, S. Hansen, V. Lynggaard, M. Ciudad, R. Vettus, P. Muda, A. Maestre, S. Castaño, S. Cheggour, J. Poulard, V. Mouquet, S. Leparrée, J. Bouet, J. Taieb, A. Doucy, H. Duquenne, A. Furber, J. Dupuis, J. Rautureau, M. Font, P. Damiano, M. Lacrimini, J. Abalea, S. Boismal, T. Menez, J. Mansourati, G. Range, H. Gorka, C. Laure, C. Vassalière, N. Elbaz, N. Lellouche, K. Djouadi, F. Roubille, D. Dietz, J. Davy, M. Granier, P. Winum, C. Leperchois-Jacquey, H. Kassim, E. Marijon, J. Le Heuzey, J. Fedida, C. Maupain, C. Himbert, E. Gandjbakhch, F. Hidden-Lucet, G. Duthoit, N. Badenco, T. Chastre, X. Waintraub, M. Oudihat, J. Lacoste, C. Stephan, H. Bader, N. Delarche, L. Giry, D. Arnaud, C. Lopez, F. Boury, I. Brunello, M. Lefèvre, R. Mingam, M. Haissaguerre, M. Le Bidan, D. Pavin, V. Le Moal, C. Leclercq, O. Piot, T. Beitar, I. Martel, A. Schmid, N. Sadki, C. Romeyer-Bouchard, A. Da Costa, I. Arnault, M. Boyer, C. Piat, N. Lozance, S. Nastevska, A. Doneva, B. Fortomaroska Milevska, B. Sheshoski, K. Petroska, N. Taneska, N. Bakrecheski, K. Lazarovska, S. Jovevska, V. Ristovski, A. Antovski, E. Lazarova, I. Kotlar, J. Taleski, L. Poposka, S. Kedev, N. Zlatanovik, S. Jordanova, T. Bajraktarova Proseva, S. Doncovska, D. Maisuradze, A. Esakia, E. Sagirashvili, K. Lartsuliani, N. Natelashvili, N. Gumberidze, R. Gvenetadze, K. Etsadashvili, N. Gotonelia, N. Kuridze, G. Papiashvili, I. Menabde, S. Glöggler, A. Napp, C. Lebherz, H. Romero, K. Schmitz, M. Berger, M. Zink, S. Köster, J. Sachse, E. Vonderhagen, G. Soiron, K. Mischke, R. Reith, M. Schneider, W. Rieker, D. Boscher, A. Taschareck, A. Beer, D. Oster, O. Ritter, J. Adamczewski, S. Walter, A. Frommhold, E. Luckner, J. Richter, M. Schellner, S. Landgraf, S. Bartholome, R. Naumann, J. Schoeler, D. Westermeier, F. William, K. Wilhelm, M. Maerkl, R. Oekinghaus, M. Denart, M. Kriete, U. Tebbe, T. Scheibner, M. Gruber, A. Gerlach, C. Beckendorf, L. Anneken, M. Arnold, S. Lengerer, Z. Bal, C. Uecker, H. Förtsch, S. Fechner, V. Mages, E. Martens, H. Methe, T. Schmidt, B. Schaeffer, B. Hoffmann, J. Moser, K. Heitmann, S. Willems, S. Willems, C. Klaus, I. Lange, M. Durak, E. Esen, F. Mibach, H. Mibach, A. Utech, M. Gabelmann, R. Stumm, V. Ländle, C. Gartner, C. Goerg, N. Kaul, S. Messer, D. Burkhardt, C. Sander, R. Orthen, S. Kaes, A. Baumer, F. Dodos, A. Barth, G. Schaeffer, J. Gaertner, J. Winkler, A. Fahrig, J. Aring, I. Wenzel, S. Steiner, A. Kliesch, E. Kratz, K. Winter, P. Schneider, A. Haag, I. Mutscher, R. Bosch, J. Taggeselle, S. Meixner, A. Schnabel, A. Shamalla, H. Hötz, A. Korinth, C. Rheinert, G. Mehltretter, B. Schön, N. Schön, A. Starflinger, E. Englmann, G. Baytok, T. Laschinger, G. Ritscher, A. Gerth, D. Dechering, L. Eckardt, M. Kuhlmann, N. Proskynitopoulos, J. Brunn, K. Foth, C. Axthelm, H. Hohensee, K. Eberhard, S. Turbanisch, N. Hassler, A. Koestler, G. Stenzel, D. Kschiwan, M. Schwefer, S. Neiner, S. Hettwer, M. Haeussler-Schuchardt, R. Degenhardt, S. Sennhenn, S. Steiner, M. Brendel, A. Stoehr, W. Widjaja, S. Loehndorf, A. Logemann, J. Hoskamp, J. Grundt, M. Block, R. Ulrych, A. Reithmeier, V. Panagopoulos, C. Martignani, D. Bernucci, E. Fantecchi, I. Diemberger, M. Ziacchi, M. Biffi, P. Cimaglia, J. Frisoni, I. Giannini, S. Boni, S. Fumagalli, S. Pupo, A. Di Chiara, P. Mirone, E. Fantecchi, F. Pesce, C. Zoccali, V. L. Malavasi, A. Mussagaliyeva, B. Ahyt, Z. Salihova, K. Koshum-Bayeva, A. Kerimkulova, A. Bairamukova, E. Mirrakhimov, B. Lurina, R. Zuzans, S. Jegere, I. Mintale, K. Kupics, K. Jubele, A. Erglis, O. Kalejs, K. Vanhear, M. Burg, M. Cachia, E. Abela, S. Warwicker, T. Tabone, R. Xuereb, D. Asanovic, D. Drakalovic, M. Vukmirovic, N. Pavlovic, L. Music, N. Bulatovic, A. Boskovic, H. Uiterwaal, N. Bijsterveld, J. De Groot, J. Neefs, N. van den Berg, F. Piersma, A. Wilde, V. Hagens, J. Van Es, J. Van Opstal, B. Van Rennes, H. Verheij, W. Breukers, G. Tjeerdsma, R. Nijmeijer, D. Wegink, R. Binnema, S. Said, Ö. Erküner, S. Philippens, W. van Doorn, H. Crijns, T. Szili-Torok, R. Bhagwandien, P. Janse, A. Muskens, M. van Eck, R. Gevers, N. van der Ven, A. Duygun, B. Rahel, J. Meeder, A. Vold, C. Holst Hansen, I. Engset, D. Atar, B. Dyduch-Fejklowicz, E. Koba, M. Cichocka, A. Sokal, A. Kubicius, E. Pruchniewicz, A. Kowalik-Sztylc, W. Czapla, I. Mróz, M. Kozlowski, T. Pawlowski, M. Tendera, A. Winiarska-Filipek, A. Fidyk, A. Slowikowski, M. Haberka, M. Lachor-Broda, M. Biedron, Z. Gasior, M. Kołodziej, M. Janion, I. Gorczyca-Michta, B. Wozakowska-Kaplon, M. Stasiak, P. Jakubowski, T. Ciurus, J. Drozdz, M. Simiera, P. Zajac, T. Wcislo, P. Zycinski, J. Kasprzak, A. Olejnik, E. Harc-Dyl, J. Miarka, M. Pasieka, M. Ziemińska-Łuć, W. Bujak, A. Śliwiński, A. Grech, J. Morka, K. Petrykowska, M. Prasał, G. Hordyński, P. Feusette, P. Lipski, A. Wester, W. Streb, J. Romanek, P. Woźniak, M. Chlebuś, P. Szafarz, W. Stanik, M. Zakrzewski, J. Kaźmierczak, A. Przybylska, E. Skorek, H. Błaszczyk, M. Stępień, S. Szabowski, W. Krysiak, M. Szymańska, J. Karasiński, J. Blicharz, M. Skura, K. Hałas, L. Michalczyk, Z. Orski, K. Krzyżanowski, A. Skrobowski, L. Zieliński, M. Tomaszewska-Kiecana, M. Dłużniewski, M. Kiliszek, M. Peller, M. Budnik, P. Balsam, G. Opolski, A. Tymińska, K. Ozierański, A. Wancerz, A. Borowiec, E. Majos, R. Dabrowski, H. Szwed, A. Musialik-Lydka, A. Leopold-Jadczyk, E. Jedrzejczyk-Patej, M. Koziel, R. Lenarczyk, M. Mazurek, Z. Kalarus, K. Krzemien-Wolska, P. Starosta, E. Nowalany-Kozielska, A. Orzechowska, M. Szpot, M. Staszel, S. Almeida, H. Pereira, L. Brandão Alves, R. Miranda, L. Ribeiro, F. Costa, F. Morgado, P. Carmo, P. Galvao Santos, R. Bernardo, P. Adragão, G. Ferreira da Silva, M. Peres, M. Alves, M. Leal, A. Cordeiro, P. Magalhães, P. Fontes, S. Leão, A. Delgado, A. Costa, B. Marmelo, B. Rodrigues, D. Moreira, J. Santos, L. Santos, A. Terchet, D. Darabantiu, S. Mercea, V. Turcin Halka, A. Pop Moldovan, A. Gabor, B. Doka, G. Catanescu, H. Rus, L. Oboroceanu, E. Bobescu, R. Popescu, A. Dan, A. Buzea, I. Daha, G. Dan, I. Neuhoff, M. Baluta, R. Ploesteanu, N. Dumitrache, M. Vintila, A. Daraban, C. Japie, E. Badila, H. Tewelde, M. Hostiuc, S. Frunza, E. Tintea, D. Bartos, A. Ciobanu, I. Popescu, N. Toma, C. Gherghinescu, D. Cretu, N. Patrascu, C. Stoicescu, C. Udroiu, G. Bicescu, V. Vintila, D. Vinereanu, M. Cinteza, R. Rimbas, M. Grecu, A. Cozma, F. Boros, M. Ille, O. Tica, R. Tor, A. Corina, A. Jeewooth, B. Maria, C. Georgiana, C. Natalia, D. Alin, D. Dinu-Andrei, M. Livia, R. Daniela, R. Larisa, S. Umaar, T. Tamara, M. Ioachim Popescu, D. Nistor, I. Sus, O. Coborosanu, N. Alina-Ramona, R. Dan, L. Petrescu, G. Ionescu, I. Popescu, C. Vacarescu, E. Goanta, M. Mangea, A. Ionac, C. Mornos, D. Cozma, S. Pescariu, E. Solodovnicova, I. Soldatova, J. Shutova, L. Tjuleneva, T. Zubova, V. Uskov, D. Obukhov, G. Rusanova, I. Soldatova, N. Isakova, S. Odinsova, T. Arhipova, E. Kazakevich, E. Serdechnaya, O. Zavyalova, T. Novikova, I. Riabaia, S. Zhigalov, E. Drozdova, I. Luchkina, Y. Monogarova, D. Hegya, L. Rodionova, L. Rodionova, V. Nevzorova, I. Soldatova, O. Lusanova, A. Arandjelovic, D. Toncev, M. Milanov, N. Sekularac, M. Zdravkovic, S. Hinic, S. Dimkovic, T. Acimovic, J. Saric, M. Polovina, B. Vujisic-Tesic, M. Nedeljkovic, M. Zlatar, M. Asanin, V. Vasic, Z. Popovic, D. Djikic, M. Sipic, V. Peric, B. Dejanovic, N. Milosevic, A. Stevanovic, A. Andric, B. Pencic, M. Pavlovic-Kleut, V. Celic, M. Pavlovic, M. Petrovic, M. Vuleta, N. Petrovic, S. Simovic, Z. Savovic, S. Milanov, G. Davidovic, V. Iric-Cupic, D. Simonovic, M. Stojanovic, S. Stojanovic, V. Mitic, V. Ilic, D. Petrovic, M. Deljanin Ilic, S. Ilic, V. Stoickov, S. Markovic, S. Kovacevic, A. García Fernandez, A. Perez Cabeza, M. Anguita, L. Tercedor Sanchez, E. Mau, J. Loayssa, M. Ayarra, M. Carpintero, I. Roldán Rabadan, M. Leal, M. Gil Ortega, A. Tello Montoliu, E. Orenes Piñero, S. Manzano Fernández, F. Marín, A. Romero Aniorte, A. Veliz Martínez, M. Quintana Giner, G. Ballesteros, M. Palacio, O. Alcalde, I. García-Bolao, V. Bertomeu Gonzalez, F. Otero-Raviña, J. García Seara, J. Gonzalez Juanatey, N. Dayal, P. Maziarski, P. Gentil-Baron, D. Shah, M. Koç, E. Onrat, I. E. Dural, K. Yilmaz, B. Özin, S. Tan Kurklu, Y. Atmaca, U. Canpolat, L. Tokgozoglu, A. K. Dolu, B. Demirtas, D. Sahin, O. Ozcan Celebi, E. Diker, G. Gagirci, U. O. Turk, H. Ari, N. Polat, N. Toprak, M. Sucu, O. Akin Serdar, A. Taha Alper, A. Kepez, Y. Yuksel, A. Uzunselvi, S. Yuksel, M. Sahin, O. Kayapinar, T. Ozcan, H. Kaya, M. B. Yilmaz, M. Kutlu, M. Demir, C. Gibbs, S. Kaminskiene, M. Bryce, A. Skinner, G. Belcher, J. Hunt, L. Stancombe, B. Holbrook, C. Peters, S. Tettersell, A. Shantsila, D. Lane, K. Senoo, K. Russell, P. Domingos, S. Hussain, J. Partridge, R. Haynes, S. Bahadur, R. Brown, S. McMahon, J. McDonald, K. Balachandran, R. Singh, S. Garg, H. Desai, K. Davies, W. Goddard, G. Galasko, I. Rahman, Y. Chua, O. Payne, S. Preston, O. Brennan, L. Pedley, C. Whiteside, C. Dickinson, J. Brown, K. Jones, L. Benham, R. Brady, L. Buchanan, A. Ashton, H. Crowther, H. Fairlamb, S. Thornthwaite, C. Relph, A. McSkeane, U. Poultney, N. Kelsall, P. Rice, T. Wilson, M. Wrigley, R. Kaba, T. Patel, E. Young, J. Law, C. Runnett, H. Thomas, H. McKie, J. Fuller, S. Pick, A. Sharp, A. Hunt, K. Thorpe, C. Hardman, E. Cusack, L. Adams, M. Hough, S. Keenan, A. Bowring, J. Watts, J. Zaman, K. Goffin, H. Nutt, Y. Beerachee, J. Featherstone, C. Mills, J. Pearson, L. Stephenson, S. Grant, A. Wilson, C. Hawksworth, I. Alam, M. Robinson, S. Ryan, R. Egdell, E. Gibson, M. Holland, D. Leonard, B. Mishra, S. Ahmad, H. Randall, J. Hill, L. Reid, M. George, S. McKinley, L. Brockway, W. Milligan, J. Sobolewska, J. Muir, L. Tuckis, L. Winstanley, P. Jacob, S. Kaye, L. Morby, A. Jan, T. Sewell, C. Boos, B. Wadams, C. Cope, P. Jefferey, N. Andrews, A. Getty, A. Suttling, C. Turner, K. Hudson, R. Austin, S. Howe, R. Iqbal, N. Gandhi, K. Brophy, P. Mirza, E. Willard, S. Collins, N. Ndlovu, E. Subkovas, V. Karthikeyan, L. Waggett, A. Wood, A. Bolger, J. Stockport, L. Evans, E. Harman, J. Starling, L. Williams, V. Saul, M. Sinha, L. Bell, S. Tudgay, S. Kemp, J. Brown, L. Frost, T. Ingram, A. Loughlin, C. Adams, M. Adams, F. Hurford, C. Owen, C. Miller, D. Donaldson, H. Tivenan, H. Button, A. Nasser, O. Jhagra, B. Stidolph, C. Brown, C. Livingstone, M. Duffy, P. Madgwick, P. Roberts, E. Greenwood, L. Fletcher, M. Beveridge, S. Earles, D. McKenzie, D. Beacock, M. Dayer, M. Seddon, D. Greenwell, F. Luxton, F. Venn, H. Mills, J. Rewbury, K. James, K. Roberts, L. Tonks, D. Felmeden, W. Taggu, A. Summerhayes, D. Hughes, J. Sutton, L. Felmeden, M. Khan, E. Walker, L. Norris, L. O’Donohoe, A. Mozid, H. Dymond, H. Lloyd-Jones, G. Saunders, D. Simmons, D. Coles, D. Cotterill, S. Beech, S. Kidd, B. Wrigley, S. Petkar, A. Smallwood, R. Jones, E. Radford, S. Milgate, S. Metherell, V. Cottam, C. Buckley, A. Broadley, D. Wood, J. Allison, K. Rennie, L. Balian, L. Howard, L. Pippard, S. Board, T. Pitt-Kerby

**Affiliations:** 1 Liverpool Centre for Cardiovascular Science, University of Liverpool and Liverpool Heart & Chest Hospital, William Henry Duncan Building, 6 West Derby St, Liverpool, L7 8TX UK; 2 Department of Translational and Precision Medicine, Sapienza University of Rome, Rome, Italy; 3 Department of Clinical Sciences and Community Health, University of Milan, Milan, Italy; 4 Geriatric Unit, IRCCS Istituti Clinici Scientifici Maugeri, Milan, Italy; 5 Cardiology Division, Department of Biomedical, Metabolic and Neural Sciences, University of Modena and Reggio Emilia, Policlinico di Modena, Modena, Italy; 6 Clinical and Experimental Medicine PhD Program, University of Modena and Reggio Emilia, Modena, Italy; 7 Service de Cardiologie, Centre Hospitalier Universitaire Trousseau, Tours, France; 8 Department of Cardiology, Hospital Universitario Virgen de la Arrixaca, IMIB-Arrixaca, University of Murcia, CIBER-CV, Murcia, Spain; 9 Department of Cardiology, Ludwig-Maximilians-University, Munich, Germany; 10 University of Medicine, ‘Carol Davila’, Colentina University Hospital, Bucharest, Romania; 11 School of Medicine, University of Belgrade, Belgrade, Serbia; 12 Intensive Arrhythmia Care, Cardiology Clinic, Clinical Center of Serbia, Belgrade, Serbia; 13 Department of Clinical Medicine, Aalborg University, Aalborg, Denmark

**Keywords:** Atrial fibrillation, Integrated management, Outcomes, Clinical complexity

## Abstract

**Background:**

Clinical complexity is increasingly prevalent among patients with atrial fibrillation (AF). The ‘Atrial fibrillation Better Care’ (ABC) pathway approach has been proposed to streamline a more holistic and integrated approach to AF care; however, there are limited data on its usefulness among clinically complex patients. We aim to determine the impact of ABC pathway in a contemporary cohort of clinically complex AF patients.

**Methods:**

From the ESC-EHRA EORP-AF General Long-Term Registry, we analysed clinically complex AF patients, defined as the presence of frailty, multimorbidity and/or polypharmacy. A K-medoids cluster analysis was performed to identify different groups of clinical complexity. The impact of an ABC-adherent approach on major outcomes was analysed through Cox-regression analyses and delay of event (DoE) analyses.

**Results:**

Among 9966 AF patients included, 8289 (83.1%) were clinically complex. Adherence to the ABC pathway in the clinically complex group reduced the risk of all-cause death (adjusted HR [aHR]: 0.72, 95%CI 0.58–0.91), major adverse cardiovascular events (MACEs; aHR: 0.68, 95%CI 0.52–0.87) and composite outcome (aHR: 0.70, 95%CI: 0.58–0.85). Adherence to the ABC pathway was associated with a significant reduction in the risk of death (aHR: 0.74, 95%CI 0.56–0.98) and composite outcome (aHR: 0.76, 95%CI 0.60–0.96) also in the high-complexity cluster; similar trends were observed for MACEs. In DoE analyses, an ABC-adherent approach resulted in significant gains in event-free survival for all the outcomes investigated in clinically complex patients. Based on absolute risk reduction at 1 year of follow-up, the number needed to treat for ABC pathway adherence was 24 for all-cause death, 31 for MACEs and 20 for the composite outcome.

**Conclusions:**

An ABC-adherent approach reduces the risk of major outcomes in clinically complex AF patients. Ensuring adherence to the ABC pathway is essential to improve clinical outcomes among clinically complex AF patients.

**Supplementary Information:**

The online version contains supplementary material available at 10.1186/s12916-022-02526-7.

## Background

In recent years, increasing awareness of clinical complexity has contributed to significant changes in the approach to the care of atrial fibrillation (AF) patients. Multimorbidity, polypharmacy and frailty can be seen as three different expressions of clinical complexity, and although these often coexist and overlap, each of these phenomena has a specific role in influencing prognosis [1]. All of these have been repeatedly described in AF patients, and several reports have outlined their detrimental effects in terms of quality of care and major outcomes [2–6].

Recent guidelines on AF [7, 8] have recommended appropriate characterization and evaluation of patients [9], followed by a holistic or integrated care approach to AF management, based on the ‘Atrial fibrillation Better Care’ (ABC) pathway approach to streamline a comprehensive and holistic management of AF patients [10]. The ABC pathway approach has three pillars: ‘A’, Anticoagulation/avoid stroke; ‘B’, Better symptom management; and ‘C’, Cardiovascular risk factors and Comorbidities optimization [10]. Adherence to the ABC pathway in patients with AF is associated with a lower risk of major outcomes and health-related costs in real-world observational studies [11–15], confirmed by the prospective mAFA-II randomized controlled trial [16] and highlighted in a systematic review and meta-analysis [17]. The latter showed that adherence to an ABC pathway was associated with lower risk of all major outcomes (including all-cause death, stroke, and major bleeding) among AF patients.

To date, there are few contemporary data on the effectiveness of the ABC pathway in specific high-risk subgroups of AF patients, particularly in clinically complex subjects. In this analysis from the European Society of Cardiology (ESC) EURObservational Research Programme (EORP) Atrial Fibrillation General Long-Term Registry, we explored whether adherence to the ABC management strategy would be associated with reduced risk of adverse outcomes in clinically complex patients, defined as those with frailty, multimorbidity and/or polypharmacy.

## Methods

For the purpose of this analysis, we used data from the ESC-EHRA EURObservational Research Programme (EORP) Atrial Fibrillation General Long-Term Registry, which is a prospective, observational, multicentre registry, held by the ESC and endorsed by the EHRA. The study enrolled consecutive AF inpatients and outpatients in 250 cardiology practices, across 27 countries. Details on study design, baseline characteristics, outcomes adjudication and follow-up are reported elsewhere [18, 19].

Briefly, all patients enrolled had AF, documented in the 12 months preceding enrolment. All patients were aged ≥18 years and provided written informed consent. Enrolment was undertaken from October 2013 to September 2016, with planned 1-year and 2-year follow-up. Patient data were collected after the signing of a written informed consent by each patient, and following the approval of the study protocol by an Institutional Review Board/Ethic Committee. The study was first approved by the National Coordinators’ main institutions (listed in the Acknowledgements section) and subsequently authorized by each site under the responsibility of the lead contact and study team (all listed in the Acknowledgements section), as per the specific national and local regulation. Any details regarding approval numbers for the study protocol regarding any specific site could be obtained from the corresponding authors, upon reasonable request. The study was performed according to the European Union Note for Guidance on Good Clinical Practice CPMP/ECH/135/95 and the Declaration of Helsinki.

Symptomatic status was defined according to EHRA score [8], while thromboembolic and bleeding risk were assessed according to CHA_2_DS_2_-VASc and HAS-BLED scores, computed according to the original schemes [8]. We defined high thromboembolic risk when CHA_2_DS_2_-VASc was ≥2 in males and ≥3 in females, and high bleeding risk when HAS-BLED was ≥3. Frailty was assessed according to a 40-item frailty index (FI) (Additional file 1: Table S1), built according to the cumulative deficits model, as proposed by Rockwood and Mitnitski [20, 21]. Calculation of FI was performed as the ratio of the total deficits found for each patient over the total number of possible deficits examined. According to the usual clinical use, a FI ranging from 0.10 to <0.25 defined the presence of pre-frailty, while a FI ≥0.25 defined the presence of frailty [22]. Multimorbidity was defined as the presence of ≥2 comorbidities. Number of drugs received at baseline was used to assess polypharmacy, which was defined as the concomitant use of ≥5 drugs [23].

Adherence to the Atrial fibrillation Better Care (ABC) pathway was evaluated at baseline and defined as per previously published study [14] according to three criteria [10]:

- *‘A’ Criterion:* Patients were considered ‘adherent’ to the ‘A’ criterion if properly prescribed with oral anticoagulant (OAC) according to their thromboembolic risk. Specifically, we considered adherent males with CHA_2_DS_2_-VASc≥1 and females with CHA_2_DS_2_-VASc≥2, treated with either vitamin K antagonist (VKA) (with a time in therapeutic range ≥70%) or a non-vitamin K antagonist oral anticoagulant (NOAC); patients not receiving OAC and with low thromboembolic risk (i.e. CHA_2_DS_2_-VASc=0 in males or =1 in females) were also considered adherent.
- *‘B’ Criterion:* As this criterion refers to the *actual* symptom control, rather than the *attempt*, we considered ‘adherent’ those patients with an EHRA score of I (no symptoms) or II (mild symptoms) at baseline.
- *‘C’ Criterion:* For this criterion, we considered the comorbidities most frequently found in AF patients: hypertension, coronary artery disease (CAD), peripheral artery disease (PAD), heart failure (HF), previous stroke/transient ischaemic attack (TIA) and diabetes mellitus. Any patient with ≥1 of these conditions and treated according to ‘optimal medical treatment’ (defined according to the current clinical guidelines) was considered adherent to this criteria. Optimal treatment was defined as follows: (i) hypertension: if blood pressure at baseline was ≤140/90 mmHg; (ii) CAD: treatment with angiotensin-converting enzyme (ACE) inhibitors, beta-blockers and statins; (iii) PAD: treatment with statins; (iv) previous stroke/TIA: treatment with statins; (v) HF: treatment with ACE inhibitors or angiotensin receptor blockers, and beta-blockers; and (vi) diabetes mellitus: treatment with insulin or oral antidiabetics. Patients with 2 or more of the above conditions needed to be optimally treated for all to be considered adherent to the ‘C’ criterion.

Patients who met all three criteria were considered adherent to the ABC pathway; otherwise, they were considered ABC-non adherent.

### Major adverse events

For this analysis, we considered the following major adverse events: (i) all-cause death; (ii) major adverse cardiovascular events (MACEs), as the composite of any thromboembolic events, any acute coronary syndrome and cardiovascular death; and (iii) a composite outcome of all-cause death and MACE.

### Statistical analysis

Continuous variables were expressed as mean (Standard Deviation, SD) or median [interquartile range, IQR]; differences across groups were evaluated with appropriate parametric and non-parametric tests, respectively. Categorical variables were expressed as counts and percentages; differences across groups were assessed through chi-square test.

Cox regression models were fitted to evaluate the impact of ABC pathway (in terms of full adherence, number of criteria fulfilled and each additional criteria) in clinically complex patients (defined as having at least one complexity criteria among frailty, multimorbidity and polypharmacy), and separately in those with frailty, multimorbidity and polypharmacy, after adjustment for age, sex, type of atrial fibrillation and components of CHA_2_DS_2_-VASc (previous thromboembolism, coronary artery disease, congestive heart failure, hypertension, diabetes, peripheral artery disease).

To explore the interplay between the different domains of clinical complexity (i.e. frailty, multimorbidity, polypharmacy), the ABC pathway and the risk of outcomes, we performed a K-medoids *cluster analysis* with the use of the partition around medoids cluster algorithm. Optimal number of clusters was selected according to the average silhouette width. We included 4 pre-determined variables in the cluster-analysis (age, frailty index, number of comorbidities and number of drugs taken), to reflect the different domains of clinical complexity; each of these variables was scaled before clustering. For each cluster, we reported baseline characteristics and compared categorical and continuous variables as already specified. We also evaluated the impact of ABC pathway adherence in each cluster identified through Kaplan-Meier and Cox-regression analyses.

To further analyse the impact of adherence to the ABC pathway on the risk of outcomes, we performed a quantile regression to estimate the *delay of event (DoE)* [24–26] attained in the ABC-adherent group of patients at 6 months, 1 year and 2 years of follow-up. Finally, for the clinical complexity group and the high clinical complexity cluster, we calculated the *number needed to treat (NNT)* along with 95% confidence interval (CI) based on absolute risk reduction at 1 year of follow-up. A two-sided *p* < 0.05 was considered statistically significant. All analyses were performed using R 4.1.2 (R Core Team, Vienna, Austria) for Windows.

## Results

Among the 11,096 patients enrolled in the ESC-EHRA EORP-AF General Long-Term Registry, a total of 9966 (89.8%) with complete data available on FI, number of comorbidities and number of drugs received were included in this analysis.

Overall, 8289 (83.1%) patients were defined as clinically complex (presenting with at least one of frailty, multimorbidity and/or polypharmacy). Baseline characteristics of the overall cohort and of the clinically complex, frailty (*n*=2108), multimorbidity (*n*=7894) and polypharmacy (*n*=5366) subgroups are shown in Additional file 1: Table S2. Overall, higher age and burden of comorbidities were associated with each of the complexity domains explored. Among patients with complete data on ABC adherence (*n*=6091), less than one-third clinically complex patients were treated as ABC-pathway adherent, with frail individuals showing lowest figures for each ABC criterion, across the clinically complexity domains explored.

### Outcomes in clinically complex and subgroups of frail, multimorbid and polypharmacy AF patients

Outcomes according to clinical complexity status and in subgroups of frail, multimorbid and polypharmacy patients during a median follow-up of 730 [IQR: 701–749] days are reported in Additional file 1: Table S3. Clinically complex AF patients showed increased risk of all-cause death (adjusted Hazard Ratio [aHR]: 1.97, 95% CI: 1.40–2.76), MACEs (aHR: 1.49, 95% CI: 1.07–2.06) and composite outcome (aHR: 1.76, 95% CI: 1.36–2.28), after adjustment for age, sex, type of AF, anticoagulation and components of CHA_2_DS_2_-VASc score. Among the clinical complexity subgroups, the highest increase in risk for all outcomes was observed for frail patients (compared to robust ones), with a 3-fold higher risk of death and composite outcome, and more than double risk of MACEs.

Multivariate Cox regression models are summarized in Table 1. Among clinically complex patients, full adherence to the ABC pathway reduced the risk of all-cause death (aHR: 0.72, 95% CI: 0.58–0.91), MACE (aHR: 0.68, 95% CI: 0.52–0.87) and composite outcome (aHR: 0.70, 95% CI: 0.58–0.85). When compared to patients’ adherent to 0 criteria, incremental risk reductions were observed as the number of ABC-adherent criteria increased.

**Table 1:** Risk of adverse clinical events and relationship with ABC pathway in clinically complex, frail, multimorbid and polypharmacy patients (*n*=6091)

	Clinically complex ***N***=4785	Frailty ***N***=1175	Multimorbidity ***N***=4535	Polypharmacy ***N***=2934
**All-cause death**				
ABC adherent, vs. non-adherent	**0.72 [0.58–0.91]**	0.63 [0.38–1.04]	**0.71 [0.57–0.89]**	0.86 [0.66–1.11]
1 ABC criteria, vs. 0	**0.53 [0.35–0.78]**	**0.50 [0.32–0.79]**	**0.51 [0.34–0.76]**	**0.52 [0.29–0.95]**
2 ABC criteria, vs. 0	**0.34 [0.23–0.50]**	**0.37 [0.23–0.58]**	**0.32 [0.21–0.47]**	**0.39 [0.22–0.69]**
3 ABC criteria, vs. 0	**0.29 [0.19–0.44]**	**0.28 [0.15–0.53]**	**0.27 [0.18–0.42]**	**0.37 [0.20–0.68]**
For each criteria	**0.69 [0.62–0.78]**	**0.67 [0.56–0.80]**	**0.68 [0.60–0.76]**	**0.80 [0.68–0.93]**
2–3 ABC criteria, vs. 0–1	**0.56 [0.47–0.68]**	**0.61 [0.46–0.81]**	**0.55 [0.45–0.66]**	**0.68 [0.53–0.88]**
**MACE**				
ABC adherent, vs. non-adherent	**0.68 [0.52–0.87]**	0.88 [0.54–1.42]	**0.69 [0.53–0.88]**	0.80 [0.60–1.06]
1 ABC criteria, vs. 0	0.69 [0.42–1.12]	**0.57 [0.33–0.98]**	0.64 [0.39–1.06]	0.63 [0.29–1.33]
2 ABC criteria, vs. 0	**0.45 [0.28–0.74]**	**0.41 [0.23–0.71]**	**0.44 [0.27–0.71]**	0.58 [0.28–1.20]
3 ABC criteria, vs. 0	**0.36 [0.21–0.60]**	**0.44 [0.22–0.85]**	**0.35 [0.21–0.58]**	0.48 [0.23–1.02]
For each criteria	**0.71 [0.62–0.81]**	**0.75 [0.61–0.92]**	**0.71 [0.63–0.82]**	0.85 [0.72–1.00]
2–3 ABC criteria, vs. 0–1	**0.59 [0.47–0.73]**	**0.66 [0.48–0.91]**	**0.60 [0.48–0.75]**	0.83 [0.61–1.12]
**Composite outcome**				
ABC adherent, vs. non-adherent	**0.70 [0.58–0.85]**	0.75 [0.50–1.12]	**0.70 [0.58–0.85]**	0.82 [0.66–1.03]
1 ABC criteria, vs. 0	**0.65 [0.45–0.95]**	**0.62 [0.40–0.95]**	**0.61 [0.42–0.90]**	0.58 [0.33–1.00]
2 ABC criteria, vs. 0	**0.43 [0.30–0.63]**	**0.46 [0.30–0.70]**	**0.41 [0.28–0.59]**	**0.46 [0.27–0.78]**
3 ABC criteria, vs. 0	**0.35 [0.24–0.52]**	**0.41 [0.24–0.71]**	**0.33 [0.22–0.49]**	**0.41 [0.23–0.71]**
For each criteria	**0.72 [0.65–0.79]**	**0.74 [0.63–0.87]**	**0.71 [0.64–0.79]**	**0.81 [0.71–0.92]**
2–3 ABC criteria, vs. 0–1	**0.59 [0.50–0.70]**	**0.66 [0.52–0.85]**	**0.59 [0.50–0.70]**	**0.72 [0.57–0.90]**

Legend: HR [95%CI] adjusted for age, sex, type of AF, hypertension, diabetes, congestive heart failure, coronary artery disease, peripheral artery disease and previous thromboembolism

We observed consistent trends in the multimorbidity and frailty group, consistent with the primary analysis above. In frail patients, adherence to each additional criterion was associated with a significant reduction in all-cause death (HR: 0.67, 95%CI: 0.56–0.80), MACE (HR: 0.75, 95%CI 0.61–0.92) and the composite outcome (HR: 0.74, 95%CI: 0.63–0.87). In the polypharmacy group, we did not observe any statistically significant associations between complete adherence to ABC pathway and outcomes; however, patients that were adherent to at least 2 ABC criteria were at lower risk of all-cause death (HR: 0.68, 95%CI: 0.53–0.88) and the composite outcome (HR: 0.72, 95%CI 0.57–0.90), but not MACE (HR 0.83, 95%CI: 0.61–1.12).

### Cluster analysis

Using a k-medoids cluster analysis, we identified 2 as the number of optimal clusters, according to the average silhouette width method. Baseline characteristics of the population according to the cluster grouping are shown in Additional file 1: Table S4.

Cluster 1 included patients with ‘high clinical complexity’, with 45% of patients being frail (median FI: 0.24, [IQR 0.20–0.29]), while cluster 2 included patients with ‘moderate clinical complexity’, with 64% being pre-frail (median FI: 0.12, IQR [0.09–0.16]). Prevalence of multimorbidity and polypharmacy was higher in cluster 1 (number of comorbidities: median (IQR), 5 [4–6] vs. 2 [1–3], *p* < 0.001; numbers of drugs 6 [5–7] vs. 4 [3–5], *p* < 0.001). Patients in the high clinical complexity cluster were older and more likely female, with higher thromboembolic and bleeding risks, and were less ABC adherent (23.4% vs. 35.1%, *p* < 0.001).

**Fig. 1:**
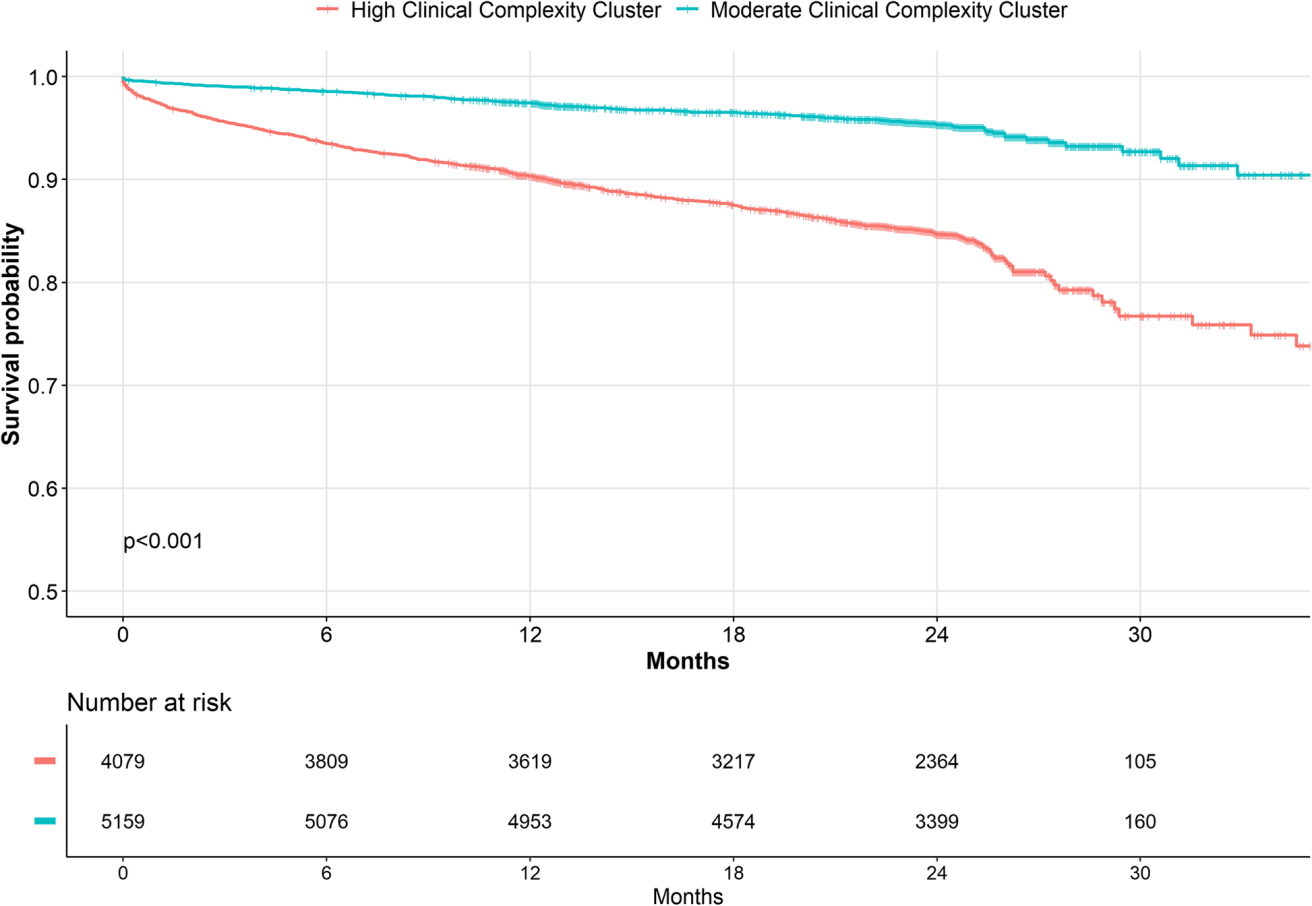
Kaplan Meier Curves for the risk of all-cause death according to cluster analysis. Legend: *p*-value for log-rank test

Kaplan-Meier curves for all-cause death, MACE and the composite outcome according to clusters are shown in Figure 1 and Additional File 1: Figure S1 and S2, respectively. Patients in the high clinical complexity cluster showed higher rates of all the outcomes investigated (*p*<0.001 for all; Table 2). Compared to the moderate complexity cluster, patients in the high clinical complexity cluster showed an increased risk of all-cause death (aHR 1.92, 95%CI 1.59–2.32), MACEs (aHR 1.95, 95%CI 1.57–2.42) and the composite outcome (aHR 1.87, 95%CI: 1.59–2.19), after adjustment for age, sex, components of CHA_2_DS_2_-VASc score, use of anticoagulant and type of AF.

**Table 2:** Risk of adverse clinical events and relationship with ABC pathway, according to cluster (*n*=6091**)**

	High clinical complexity cluster ***N***=2398	Moderate clinical complexity cluster ***N***=3693
**All-cause death,** ***n*** **(%)**	363 (15.1)	172 (4.6)
ABC adherent, vs. non-adherent	**0.74 [0.56–0.98]**	0.83 [0.59–1.16]
1 ABC criteria, vs. 0	**0.54 [0.34–0.84]**	0.42 [0.18–1.01]
2 ABC criteria, vs. 0	**0.36 [0.23–0.56]**	**0.32 [0.14–0.73]**
3 ABC criteria, vs. 0	**0.32 [0.20–0.52]**	**0.29 [0.12–0.69]**
For each criteria	**0.71 [0.62–0.82]**	**0.79 [0.64–0.96]**
2–3 ABC criteria, vs. 0–1	**0.60 [0.48–0.75]**	**0.67 [0.47–0.96]**
**MACE,** ***n*** **(%)**	375 (15.6)	203 (5.5)
ABC adherent, vs. non-adherent	0.80 [0.60–1.08]	**0.58 [0.39–0.85]**
1 ABC criteria, vs. 0	0.63 [0.36–1.09]	0.62 [0.24–1.60]
2 ABC criteria, vs. 0	**0.45 [0.26–0.78]**	0.41 [0.16–1.03]
3 ABC criteria, vs. 0	**0.41 [0.23–0.74]**	**0.27 [0.10–0.70]**
For each criteria	**0.77 [0.66–0.90]**	**0.65 [0.53–0.81]**
2–3 ABC criteria, vs. 0–1	**0.66 [0.51–0.85]**	**0.55 [0.38–0.79]**
**Composite outcome,** ***n*** **(%)**	554 (23.1)	320 (8.7)
ABC adherent, vs. non-adherent	**0.76 [0.60–0.96]**	**0.70 [0.54–0.92]**
1 ABC criteria, vs. 0	**0.62 [0.40–0.94]**	0.65 [0.29–1.42]
2 ABC criteria, vs. 0	**0.44 [0.29–0.66]**	0.46 [0.21–1.00]
3 ABC criteria, vs. 0	**0.38 [0.25–0.60]**	**0.36 [0.16–0.80]**
For each criteria	**0.75 [0.66–0.85]**	**0.74 [0.63–0.87]**
2–3 ABC criteria, vs. 0–1	**0.64 [0.53–0.78]**	**0.63 [0.48–0.84]**

Legend: HR [95%CI] adjusted for age, sex, type of AF, hypertension, diabetes, congestive heart failure, coronary artery disease, peripheral artery disease and previous thromboembolism

Cox regression analyses (Table 2) showed that in the high clinical complexity cluster, adherence to ABC pathway was associated with reduced risk of all-cause death (aHR: 0.74, 95%CI: 0.56–0.98) and the composite outcome (aHR: 0.76, 95%CI: 0.60–0.96) but not MACE. Adherence to an increasing number of ABC criteria was associated with risk reductions for all outcomes. Adherence to at least 2 ABC criteria was associated with a lower risk of all events when compared to subjects’ adherent to 0 or 1 criteria.

In the moderate complexity cluster, full ABC pathway adherence was associated with a significant reduction in the risk of MACE and composite outcome, but not all-cause death; however, adherence to at least 2 ABC criteria was associated with a significant reduction of the risk of all-cause death when compared to patients adherent to 0–1 criteria.

### Delay of event analysis and NNT

In clinically complex patients, ABC-adherent management resulted in a significant delay of all events investigated: at 1-year follow-up, ABC adherent patients gained 402 [95%CI: 242–1018] days of survival, 396 [95%CI: 57–573] days of MACE-free time and 385 [95%CI: 303–491] days of composite outcome-free time (Table 3). Based on absolute risk reduction at 1 year of follow-up, the NNTs for ABC pathway adherence was 24 (95%CI: 18–37) for all-cause death, 31 (95%CI: 22–56) for MACEs and 20 (95%CI: 15–31) for the composite outcome. Similar trends were noted in the different subgroups of frailty, multimorbidity and polypharmacy.

**Table 3:** Delay of event analysis, ABC adherent vs. non adherent (*n*=6091)

	Clinically complex ***N***=4785	Frailty ***N***=1175	Multimorbidity ***N***=4535	Polypharmacy ***N***=2934	High clinical complexity cluster ***N***=2398
**All-cause death**					
6 months	430 [284–613]	350 [142–736]	456 [297–608]	264 [77–427]	302 [117–485]
1 year	402 [242–1018]	NA	405 [232–970]	342 [204–521]	359 [211–531]
2 years	214 [40–776]	NA	214 [36–784]	296 [194–472]	318 [60–529]
**MACE**					
6 months	330 [180–464]	116 [−120 to 370]	258 [110–424]	151 [−8 to 321]	161 [31–307]
1 year	396 [57–573]	151 [−181 to 554]	395 [140–586]	317 [143–502]	275 [87–467]
2 years	197 [−301 to 395]	23 [−94 to 181]	194 [−342 to 432]	200 [55–393]	200 [−46 to 365]
**Composite outcome**					
6 months	305 [65–544]	141 [−12 to 361]	311 [31–538]	157 [29–289]	189 [70–356]
1 year	385 [303–491]	275 [−24 to 626]	384 [305–490]	310 [177–422]	324 [159–481]
2 years	194 [−181 to 426]	NA	197 [−147 to 422]	200 [24–379]	189 [36–356]

Legend: The estimates are for the delay of event in the ABC adherent subgroup, calculated at each time point and according to survival in the ABC non-adherent subgroup (and expressed as days free of the respective outcome). *NA* not available

**Fig. 2:**
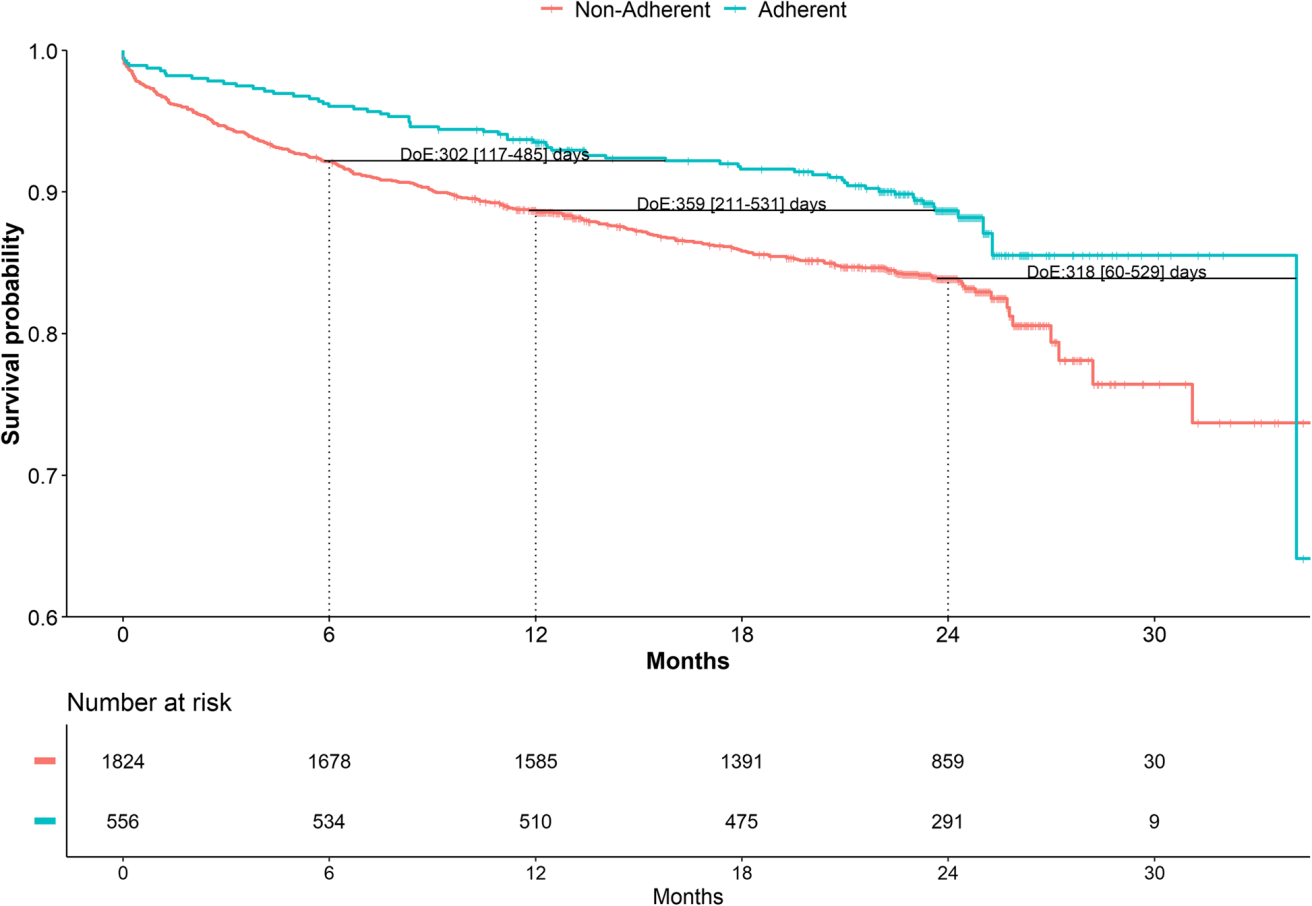
Delay of event analysis for all-cause death, ABC adherent vs. non-adherent in high-complexity cluster subgroup. Legend: DoE, delay of event. Figures reported are estimates [95% confidence intervals]

When analysing the DoEs, individuals in the high clinical complexity cluster who were adherent to the ABC-pathway showed gain of event-free survival for all the outcomes at most time-points investigated (Figure 2 for all-cause mortality and Additional File 1: Figure S3 and S4 for MACE and composite outcome, respectively). At 1 year of follow-up, the ABC-adherent NNT in the high clinical complexity cluster was 21 (95%CI: 14–44) for all-cause death, 34 (95%CI: 19–250) for MACEs, and 22 (95%CI: 13–67) for the composite outcome.

## Discussion

**Fig. 3:**
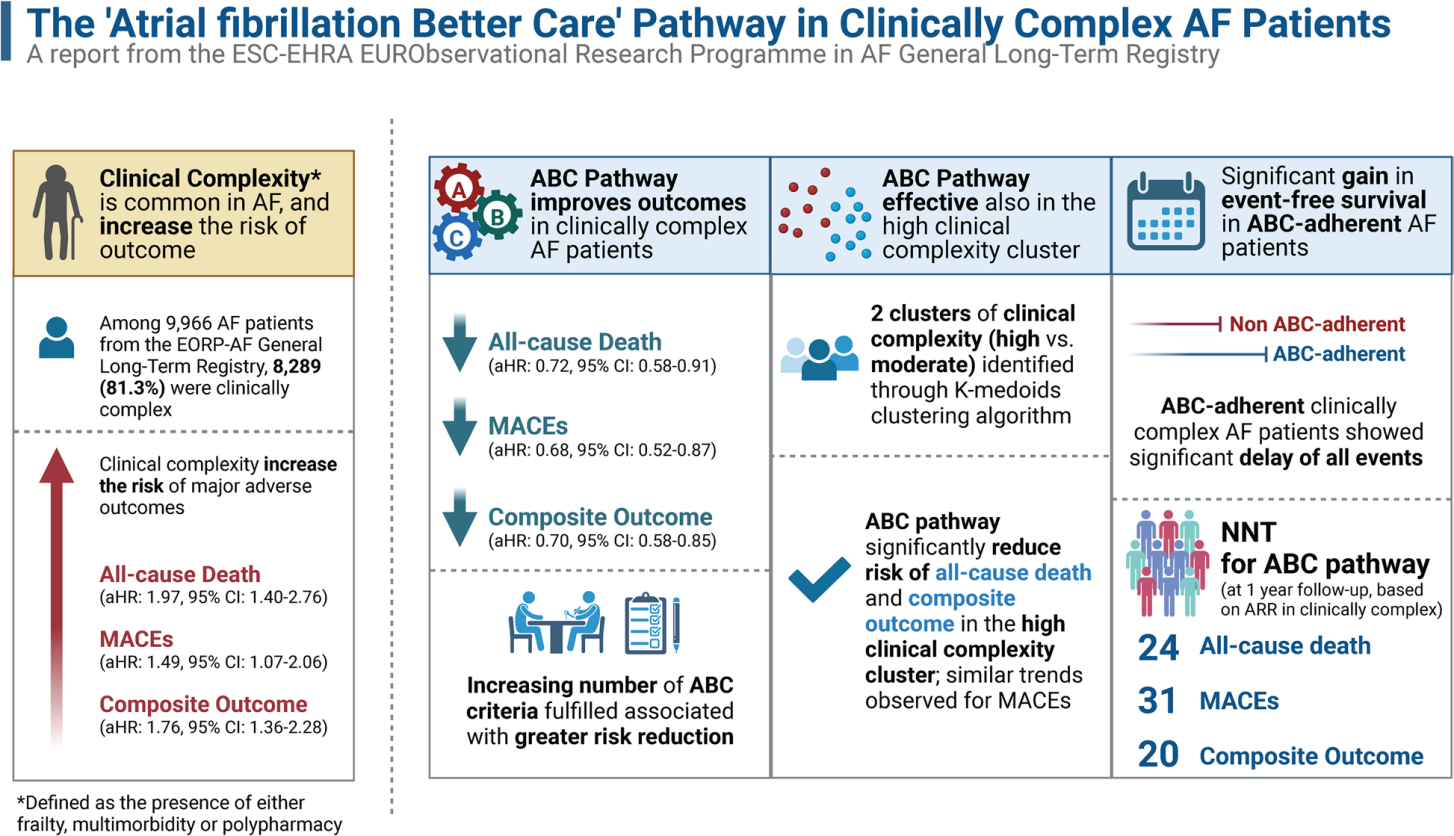
Central Illustration (Created with Biorender.com)

Our results show that clinically complex AF patients have a poor prognosis, encompassed by an increased risk of all the outcomes investigated, including death, MACE and composite outcome. Our principal findings were as follows: (i) adherence to the ABC pathway in the clinically complex group reduced the risk of all-cause death, MACE and the composite outcome; (ii) these findings were further confirmed by the cluster analysis, and the DoE data whereby an ABC-adherent approach resulted in significant gains in event-free survival for all the outcomes investigated; and (iii) the NNTs for ABC pathway adherence was 24 for all-cause death, 31 for MACEs and 20 for the composite outcome at 1 year of follow-up (Fig. 3).

In this study, we provide the first systematic assessment of the efficacy of a comprehensive and integrated approach (the ABC pathway) for the management of AF patients, in clinically complex patients. Consistent with our ‘proof of concept’ post hoc analysis from an (old) AFFIRM trial dataset [15], ABC-adherent management reduced the risk of all-cause death, MACE and composite outcome among clinically complex patients, and the magnitude of the effect increased with the number of the ABC criteria fulfilled. With the use of K-medoids cluster analysis, we were able to show the beneficial impact of the ABC adherent approach according to different levels of overall complexity, reflecting the real-world scenario of patients with interacting frailty, multimorbidity and polypharmacy. Also, using a DoE analysis approach, ABC-adherent clinically complex AF patients had a meaningful gain in event-free survival.

For our primary analysis, we defined clinical complexity according to three domains (i.e. frailty, multimorbidity and/or polypharmacy). Our manuscript is also the first to analyse the efficacy of an integrated care approach among three domains of clinical complexity, which are closely but not interchangeable [1]; these three entities capture different expressions of clinical complexity, which often coexist and act synergistically in real-world patients, leading to worse outcomes. The strength of this approach is to consider clinical complexity as a whole entity, encompassing not only the presence of multiple risk factors, but also the accumulation of deficits and the complexities arising from polypharmacy, an often-underestimated issue in AF patients.

In the real world, the presence of frailty, multimorbidity and/or polypharmacy tend to cluster, and the results of our cluster analysis are particularly important given that AF patients often present with a multifaceted interplay between different complexity criteria, leading to a synergistic detrimental effect. Indeed, the high clinical complexity cluster identified a group of AF patients (45% frail, virtually all burdened by multimorbidity, and the vast majority (83%) treated with 5 or more drugs) for whom—given the poor prognosis—there is an urgent need for effective interventions.

Management according to the ABC pathway provided approximately 30% reduction of the risk of all outcomes; the magnitude of the effect was even greater when comparing patients who were adherent to all the ABC criteria with those who were completely non-adherent, with a 65 to 71% reduction in the risk of death, MACE and composite outcome. Indeed, based on absolute risk reduction at 1 year of follow-up, the NNTs for ABC pathway adherence show how implementation of an ABC approach can avoid one death or the composite outcome in approximately every 20 patients who were clinically complex or in the high complexity cluster, while slightly higher figures were found for MACE events. The usefulness of the ABC approach is further reinforced by the DoE analysis. Indeed, an ABC-adherent approach resulted in more than 12 months delay of all outcomes in the clinically complex group at 1 year of follow-up, even in the high complexity cluster.

Our results are aligned with the findings of the main analysis on the effect of the ABC-pathway in the ESC-EHRA EORP Long Term Registry [14] and reinforce the importance of a comprehensive management in the care of clinically complex AF patients [15]. To date, awareness of the effect of frailty [5, 27], multimorbidity [4, 28, 29] and polypharmacy [3] in AF patients is increasing, with compelling evidence on their detrimental effects on quality of care, efficacy of treatment and finally adverse outcomes [2, 4, 29]. However, there is still limited data on how to handle complexity in clinical practice. In recent years, there has been a shift from the focus on single comorbidities (while important in determining the risk of outcomes in AF patients [30–32]), to that on multimorbidity in influencing clinical management and outcomes [4, 6, 29, 33]. In this real-world analysis from a large contemporary European AF cohort, we show that the main issue is not exclusively the burden of diseases (nor the impairment of functionality or the burden of medications), but the overall higher clinical complexity, irrespective of which are the main components, in directly influencing the prognosis of AF patients.

Our findings have several important clinical implications. First, clinical complexity is common among the general AF population and needs awareness and specific strategies to tackle the higher risks associated with this state. Second, the proportion of clinically complex patients adherent to the ABC pathway was unsatisfactory among all the clinical complexity domains explored, especially so in the high complexity cluster. Given the beneficial clinical outcomes with ABC pathway adherenc e[17], this should lead to proactive strategies to improve the adoption of integrated care management in these subgroups of patients, as advocated in other complex chronic long-term conditions [34, 35]. Third, adherence to the ABC pathway was effective in reducing the risk of major outcomes, with a clear trend towards greater benefit with increasing ABC criteria attained, resulting in longer event-free survival with ABC-adherence. These improvements would impact on quality of life and healthcare-associated costs, which are highly relevant given the increasing burden of AF on healthcare systems [36].

### Limitations

Our analysis has some limitations. The observational design and the limited power to evaluate subgroups that were not specified in the original study design may limit the generalizability of our findings; however, we have analysed more than 6000 patients with complete data on the ABC pathway, this contributing to the reliability of our estimates. Furthermore, we have provided extensive adjustment of our analyses on the evaluation of the effect of the ABC pathway on the risk of outcomes. While we cannot exclude the contribution of other unaccounted bias, which may have contributed to the results presented, our results are consistent with previous evidence on the effect of ABC pathway in similar scenarios, this further reinforcing our results [15, 37]. Finally, our cohort was established across European countries and may not completely reflect other AF populations; therefore, our results may not be immediately applicable to other cohorts and require further evaluation in other geographical settings.

## Conclusions

An ABC-adherent approach reduces the risk of major outcomes in AF patients characterized by clinical features of complexity (i.e. frailty, multimorbidity and polypharmacy). Ensuring adherence to the ABC pathway is essential to improve clinical outcomes among clinically complex AF patients.

## Supplementary Information

**Additional file 1: Table S1**. Items Included into the Frailty Index. **Table S2**. Baseline Characteristics of the Cohort. **Table S3**. Cox Regression for the risk of major outcomes according to clinical complexity and subgroups. **Table S4**. Baseline characteristics according to cluster allocation. **Figure S1**. Kaplan Meier Curves for the risk of MACE according to cluster analysis. **Figure S2**. Kaplan Meier Curves for the risk of composite outcome according to cluster analysis. **Figure S3**. Delay of Event analysis for MACE, ABC adherent vs. non-adherent in cluster 1 subgroup. **Figure S4**. Delay of Event analysis for Composite Outcome, ABC adherent vs. non-adherent in cluster 1 subgroup.

## Data Availability

The data that support the findings of this study are available from the corresponding author, upon reasonable request, and after approval of all other co-investigators.

## Abbreviations

ABC: Atrial fibrillation Better Care
ACE: Angiotensin-converting enzyme
AF: Atrial fibrillation
CAD: Coronary artery disease
CI: Confidence interval
DoE: Delay of event
EHRA: European Heart Rhythm Association
EORP: EURObservational Research Programme
ESC: European Society of Cardiology
FI: Frailty index
HF: Heart failure
HR: Hazard ratio
IQR: Interquartile range
MACE: Major adverse cardiovascular event
NNT: Number needed to treat
NOAC: Non-vitamin K antagonist oral anticoagulant
OAC: Oral anticoagulant
PAD: Peripheral artery disease
SD: Standard deviation
TIA: Transient ischaemic attack
VKA: Vitamin K antagonist
