# Supplementary material for: Clinical complexity and impact of the ABC (Atrial fibrillation Better Care) pathway in patients with atrial fibrillation: a report from the ESC-EHRA EURObservational Research Programme in AF General Long-Term Registry
Source: BMC Med. 2022 Sep 2;20:326. doi: 10.1186/s12916-022-02526-7 (PMC9440492; doi:10.1186/s12916-022-02526-7)

**Clinical Complexity and Impact of the ABC (Atrial fibrillation Better Care) Pathway in Patients with Atrial Fibrillation: A report from the ESC-EHRA EURObservational Research Programme in AF General Long-Term Registry**

**Brief Title:** Clinical Complexity in AF Patients

Additional File 1

| Supplementary Tables | Page 2 |
| --- | --- |
| Supplementary Figures | Page 11 |
| Appendix | Page 15 |

**Table S1: Items Included into the Frailty Index**

| ***1. Biological Parameters Domain*** | **DEFINITION** | **DEFICIT**  **VALUE** |
| --- | --- | --- |
| Systolic Blood Pressure | ≥140 mmHg | 1 |
| Diastolic Blood Pressure | ≥90 mmHg | 1 |
| Heart Rate | ≥110 bpm | 1 |
| Body Mass Index | <18.5 kg/m^2^  25.0-29.9 kg/m^2^  ≥30.0 kg/m^2^ | 1  0.5  1 |
| ***2. Comorbidities Domain*** |  |  |
| Hypertension | Present | 1 |
| Diabetes Mellitus | Present | 1 |
| Lipid Disorder | Present | 1 |
| Coronary Artery Disease | Present | 1 |
| Heart Failure | Present | 1 |
| Valvular Disease | Present | 1 |
| Cardiomyopathy | Present | 1 |
| Pulmonary Arterial Hypertension | Present | 1 |
| Peripheral Artery Disease | Present | 1 |
| Previous Thromboembolic Events | Present | 1 |
| Previous Hemorrhagic Events | Present | 1 |
| Hyperthyroidism | Present | 1 |
| Hypothyroidism | Present | 1 |
| Chronic Kidney Disease | Present | 1 |
| Liver Disease | Present | 1 |
| Chronic Obstructive Pulmonary Disease | Present | 1 |
| Obstructive Sleep Apnoea Syndrome | Present | 1 |
| Dementia | Present | 1 |
| History of Anaemia | Present | 1 |
| Malignancy | Present | 1 |
| ***3. Symptoms Domain*** |  |  |
| Palpitations | Present | 1 |
| Syncope | Present | 1 |
| Shortness of Breath | Present | 1 |
| Chest Pain | Present | 1 |
| General not-Well Being | Present | 1 |
| Dizziness | Present | 1 |
| Fatigue | Present | 1 |
| Fear | Present | 1 |
| Other Symptoms | Present | 1 |
| ***4. Function/Autonomy Domain***  *(EQ-5D-5L Questionnaire)* |  |  |
| Mobility | No Problems  Slight Problems  Moderate Problems  Severe Problems  Unable to Walk | 0  0.25  0.5  0.75  1 |
| Self-Care | No Problems  Slight Problems  Moderate Problems  Severe Problems  Unable to Wash/Dress | 0  0.25  0.5  0.75  1 |
| Usual Activities | No Problems  Slight Problems  Moderate Problems  Severe Problems  Unable to Usual Activities | 0  0.25  0.5  0.75  1 |
| Pain/Discomfort | No Pain/Discomfort  Slight Pain/Discomfort  Moderate Pain/Discomfort  Severe Pain/Discomfort  Extreme Pain/Discomfort | 0  0.25  0.5  0.75  1 |
| Anxiety/Depression | None  Slight Anxious/Depressed  Moderate Anxious/Depressed  Severely Anxious/Depressed  Extremely Anxious/Depressed | 0  0.25  0.5  0.75  1 |
| ***5. Biomarkers Domain*** |  |  |
| Creatinine Clearance (CKD-EPI) | <60 mL/min | 1 |
| Haemoglobin | <13 mg/dL for males  <12 mg/dL for females | 1 |

**Table S2: Baseline Characteristics of the Cohort**

| **Variables** | **Total N=9966** | **Non Clinically Complex  N=1677** | **Clinically Complex N=8289** | **Frailty**  **N=2108** | **Multimorbidity  N=7894** | **Polypharmacy N=5366** |
| --- | --- | --- | --- | --- | --- | --- |
| *Socio-Demographic Characteristics* | |  |  |  |  |  |
| **Age**, *years* median [IQR] | 71 [62-77] | 63 [53-72] | 72 [64-78] | 73 [66-79] | 72 [64-78] | 72 [65-78] |
| **Female**, n (%) | 4004 (40.2) | 549 (32.7) | 3455 (41.7) | 1038 (49.2) | 3301 (41.8) | 2285 (42.6) |
| **European Region**, n (%)  Northern Europe  Western Europe  Eastern Europe  Southern Europe | 1331 (13.4) 3242 (32.5) 1643 (16.5) 3750 (37.6) | 412 (24.6) 553 (33.0) 141 (8.4) 571 (34.0) | 919 (11.1) 2689 (32.4) 1502 (18.1) 3179 (38.4) | 191 (9.1) 481 (22.8) 593 (28.1) 843 (40.0) | 827 (10.5) 2567 (32.5) 1447 (18.3) 3053 (38.7) | 546 (10.2) 1631 (30.4) 1008 (18.8) 2181 (40.6) |
| *Clinical Characteristics and Comorbidities* | |  |  |  |  |  |
| **Site of Inclusion**, n (%)  Outpatient Facility  Hospital | 4785 (48.0) 5181 (52.0) | 935 (55.8) 742 (44.2) | 3850 (46.4) 4439 (53.6) | 632 (30.0) 1476 (70.0) | 3664 (46.4) 4230 (53.6) | 2208 (41.1) 3158 (58.8) |
| **Reason for Admission**, n (%)  Other than AF  AF | 3360 (33.7) 6606 (66.3) | 172 (10.3) 1505 (89.7) | 3188 (38.5) 5101 (61.5) | 1015 (48.1) 1093 (51.9) | 3114 (39.4) 4780 (60.6) | 2356 (43.9) 3010 (56.1) |
| **BMI**, *kg/m^2^* median [IQR] | 27.5 [24.8-31.1] | 26.7 [24.4-30.1] | 27.7 [24.8-31.2] | 28.8 [25.5-32.7] | 27.7 [24.8-31.2] | 28.0 [25.2-31.6] |
| **SBP**, *mmHg* median [IQR] | 130 [120-142] | 130 [120-140] | 130 [120-144] | 140 [120-150] | 130 [120-144] | 130 [120-145] |
| **DBP**, *mmHg* median [IQR] | 80 [70-87] | 80 [70-86] | 80 [70-88] | 80 [70-90] | 80 [70-87] | 80 [70-87] |
| **LVEF,** *%* median [IQR] | 55 [45-61] | 60 [55-64] | 55 [45-60] | 50 [39-60] | 55 [45-60] | 53 [40-60] |
| **Type of AF**, n (%)  First Diagnosed  Paroxysmal Persistent Long-Standing Persistent Permanent Unknown | 1583 (15.9) 2612 (26.2) 1962 (19.7) 429 (4.3) 3220 (32.3)  157 (1.6) | 379 (22.6)  602 (35.9)  373 (22.2)  49 (2.9)  230 (13.7)  44 (2.6) | 1204 (14.5)  2010 (24.3)  1589 (19.2)  380 (4.6)  2990 (36.1)  113 (1.4) | 315 (15.0)  485 (24.0)  384 (18.2)  110 (5.2)  794 (37.7)  18 (0.9) | 1120 (14.2)  1904 (24.1)  1488 (18.9)  361 (4.6)  2912 (36.9)  107 (1.4) | 749 (14.0)  1254 (23.4)  997 (18.6)  246 (4.6)  2055 (38.3)  65 (1.2) |
| **Heart Failure**, n (%) | 3793 (38.1) | 35 (2.1) | 3758 (45.3) | 1539 (73.0) | 3726 (47.2) | 2721 (50.7) |
| **Coronary Artery Disease**, n (%) | 2773 (27.8) | 31 (1.8) | 2742 (33.1) | 992 (47.1) | 2709 (34.3) | 2093 (39.0) |
| **Hypertension**, n (%) | 6168 (61.9) | 349 (20.8) | 5819 (70.2) | 1701 (80.7) | 5684 (72.0) | 3870 (72.1) |
| **Diabetes Mellitus**, n (%) | 2277 (22.8) | 34 (2.0) | 2243 (27.1) | 914 (43.4) | 2225 (28.2) | 1858 (34.6) |
| **Lipid Disorder**, n (%) | 3970 (39.8) | 132 (7.9) | 3838 (46.3) | 1212 (57.5) | 3804 (48.2) | 2679 (49.9) |
| **Previous TE Events**, n (%) | 1150 (11.5) | 83 (4.9) | 1067 (12.9) | 398 (18.9) | 1041 (13.2) | 714 (13.3) |
| **Previous Hemorrhagic Events**, n (%) | 527 (5.3) | 48 (2.9) | 479 (5.8) | 214 (10.2) | 466 (5.9) | 333 (6.2) |
| **PAD**, n (%) | 792 (7.9) | 11 (0.7) | 781 (9.4) | 378 (17.9) | 780 (9.9) | 570 (10.6) |
| **CKD**, n (%) | 1194 (12.0) | 11 (0.7) | 1183 (14.4) | 649 (30.9) | 1179 (15.0) | 889 (16.7) |
| **COPD**, n (%) | 889 (8.9) | 21 (1.3) | 868 (10.5) | 395 (18.7) | 865 (11.0) | 655 (12.2) |
| **Anaemia**, n (%) | 521 (5.2) | 4 (0.2) | 517 (6.2) | 327 (15.5) | 515 (6.5) | 350 (6.5) |
| **Dementia**, n (%) | 110 (1.1) | 1 (0.1) | 109 (1.3) | 68 (3.2) | 109 (1.4) | 66 (1.2) |
| **Malignancy**, n (%) | 741 (7.4) | 36 (2.2) | 705 (8.5) | 230 (10.9) | 700 (8.8) | 405 (7.6) |
| **CHA_2_DS_2_-VASc**, median [IQR] | 3 [2-4] | 1 [0-2] | 3 [2-5] | 4 [3-5] | 4 [2-5] | 4 [3-5] |
| **High TE Risk**, n (%) | 7490 (75.2) | 514 (30.7) | 6976 (84.2) | 1994 (94.6) | 6777 (85.9) | 4713 (87.9) |
| **HAS-BLED**, Median [IQR] | 1 [1-2] | 1 [0-1] | 2 [1-2] | 2 [1-3] | 2 [1-2] | 2 [1-2] |
| **High Bleeding Risk**, n (%) | 1723 (17.3) | 53 (3.2) | 1670 (20.1) | 750 (35.6) | 1637 (20.7) | 1210 (22.5) |
| *ABC Criteria (n=6091)* |  |  |  |  |  |  |
| **ABC compliant,** n (%) | 1856 (30.5) | 461 (35.3) | 1395 (29.2) | 143 (12.2) | 1299 (28.6) | 931 (31.7) |
| **Number of ABC criteria fulfilled**, n (%)  0 1 2 3 | 133 (2.2) 1139 (18.7) 2963 (48.6) 1856 (30.5) | 18 (1.4) 210 (16.1) 617 (47.2) 461 (35.3) | 115 (2.4) 929 (19.4) 2346 (49.0) 1395 (29.2) | 80 (6.8) 394 (33.5) 558 (47.5) 143 (12.2) | 108 (2.4) 880 (19.4) 2248 (49.6) 1299 (28.7) | 50 (1.7) 497 (16.9) 1456 (49.6) 931 (31.7) |
| **‘A’ criterion adherent,** n (%) | 4624 (75.9) | 808 (61.9) | 3816 (79.7) | 890 (75.7) | 3634 (80.1) | 2500 (85.2) |
| **‘B’ criterion adherent,** n (%) | 4890 (80.3) | 1100 (84.2) | 3790 (79.2) | 681 (58.0) | 3600 (79.4) | 2283 (77.8) |
| **‘C’ criterion adherent,** n (%) | 3119 (51.2) | 919 (70.4) | 2200 (46.0) | 368 (31.3) | 2039 (45.0) | 1419 (48.4) |

Legend: AF: Atrial Fibrillation; BMI: Body Mass Index; CKD: Chronic Kidney Disease; COPD: Chronic Obstructive Pulmonary Disease; DBP: Diastolic Blood Pressure; LVEF: Left Ventricular Ejection Fraction; IQR: Interquartile Range; PAD: Peripheral Artery Disease; SBP: Systolic Blood Pressure; TE: Thromboembolism

**Table S3 - Cox Regression for the risk of major outcomes according to clinical complexity and subgroups**

|  | **Clinically Complex**  **N=8289** | **Frailty N=2108** | **Multimorbidity N=7894** | **Polypharmacy N=5366** |
| --- | --- | --- | --- | --- |
| **All-Cause Death, n (%)** | 848 (10.2) | 350 (16.6) | 829 (10.5) | 592 (11.0) |
| *aHR [95% CI]** | **1.97 [1.40-2.76]** | **3.10 [2.23-4.29]**^†^ | **1.69 [1.26-2.27]** | **1.23 [1.05-1.44]** |
| **MACEs, n (%)** | 840 (10.1) | 348 (16.5) | 824 (10.4) | 637 (11.9) |
| *aHR [95% CI]** | **1.49 [1.07-2.06]** | **2.49 [1.77-3.50]**^†^ | **1.53 [1.13-2.08]** | **1.42 [1.18-1.70]** |
| **Composite Outcome, n (%)** | 1311 (15.8) | 522 (24.8) | 1278 (16.2) | 939 (17.5) |
| *aHR [95% CI]** | **1.76 [1.36-2.28]** | **2.95 [2.27-3.83]**^†^ | **1.60 [1.27-2.02]** | **1.25 [1.10-1.44]** |

**Legend:** aHR= Adjusted Hazard Ratio. CI= Confidence Intervals. MACEs= Major Adverse Cardiovascular Events. *adjusted for: age, sex, hypertension, congestive heart failure, coronary artery disease, previous thromboembolism, peripheral artery disease, type of AF, use of anticoagulant. ^†^As compared with robust.

**Table S4: Baseline characteristics according to cluster allocation**

| **Variables** | **High Clinical Complexity Cluster**  **N= 4437** | **Moderate Clinical Complexity Cluster  N= 5529** | **P** |
| --- | --- | --- | --- |
| *Cluster Variables* |  |  |  |
| **Frailty Status**, n (%)  Robust Pre-Frail Frail | 2 (0.0) 2428 (54.7) 2007 (45.2) | 1905 (34.5) 3523 (63.7) 101 (1.8) | <0.001 |
| **Frailty Index**, median [IQR] | 0.24 [0.20-0.29] | 0.12 [0.09-0.16] | <0.001 |
| **Multimorbidity**, n (%) | 4417 (99.5) | 3477 (62.9) | <0.001 |
| **Number of comorbidities,** median [IQR] | 5 [4-6] | 2 [1-3] |  |
| **Polypharmacy**, n (%) | 3693 (83.2) | 1673 (30.3) | <0.001 |
| **Number of drugs**, median [IQR] | 6 [5-7] | 4 [3-5] | <0.001 |
| *Socio-Demographic Characteristics* |  |  |  |
| **Age**, *years* median [IQR] | 74 [67-79] | 67 [59-75] | <0.001 |
| **Female**, n (%) | 1979 (44.6) | 2025 (36.6) | <0.001 |
| **European Region**, n (%)  Northern Europe  Western Europe  Eastern Europe  Southern Europe | 373 (8.4) 1337 (30.1) 985 (22.2) 1742 (39.3) | 958 (17.3) 1905 (34.5) 658 (11.9) 2008 (36.3) | <0.001 |
| *Clinical Characteristics and Comorbidities* | |  |  |
| **Site of Inclusion**, n (%)  Outpatient Facility  Hospital | 1708 (38.5) 2729 (61.5) | 3077 (55.7) 2452 (44.3) | <0.001 |
| **Reason for Admission**, n (%)  Other than AF  AF | 2184 (49.2) 2253 (50.8) | 1176 (21.3) 4353 (78.7) | <0.001 |
| **BMI**, *kg/m^2^* median [IQR] | 28.1 [25.2-31.8] | 27.1 [24.5-30.4] | <0.001 |
| **SBP**, *mmHg* median [IQR] | 130 [120-145] | 130 [120-140] | <0.001 |
| **DBP**, *mmHg* median [IQR] | 80 [70-89] | 80 [70-86] | 0.051 |
| **LVEF,** *%* median [IQR] | 51 [40-60] | 59 [50-63] | <0.001 |
| **Type of AF**, n (%)  First Diagnosed  Paroxysmal Persistent Long-Standing Persistent Permanent Unknown | 576 (13.0)  974 (22.0)  736 (16.6)  211 (4.8)  1888 (42.6)  51 (1.1) | 1007 (18.2)  1638 (29.6)  1226 (22.2)  218 (3.9)  1332 (24.1)  106 (1.9) | <0.001 |
| **Heart Failure**, n (%) | 2833 (63.8) | 960 (17.4) | <0.001 |
| **Coronary Artery Disease**, n (%) | 2048 (46.2) | 725 (13.1) | <0.001 |
| **Hypertension**, n (%) | 3492 (78.7) | 2676 (48.4) | <0.001 |
| **Diabetes Mellitus**, n (%) | 1766 (39.8) | 511 (9.2) | <0.001 |
| **Lipid Disorder**, n (%) | 2525 (56.9) | 1445 (26.1) | <0.001 |
| **Previous TE Events**, n (%) | 698 (15.7) | 452 (8.2) | <0.001 |
| **Previous Hemorrhagic Events**, n (%) | 352 (7.9) | 175 (3.2) | <0.001 |
| **PAD**, n (%) | 661 (14.9) | 131 (2.4) | <0.001 |
| **CKD**, n (%) | 1017 (23.1) | 177 (3.0) | <0.001 |
| **COPD**, n (%) | 679 (15.3) | 210 (3.8) | <0.001 |
| **Anaemia**, n (%) | 460 (10.4) | 61 (1.1) | <0.001 |
| **Dementia**, n (%) | 97 (2.2) | 13 (0.2) | <0.001 |
| **Malignancy**, n (%) | 469 (10.6) | 272 (4.9) | <0.001 |
| **CHA_2_DS_2_-VASc**, median [IQR] | 4 [3-5] | 2 [1-3] | <0.001 |
| **High TE Risk**, n (%) | 4246 (95.8) | 3244 (58.7) | <0.001 |
| **HAS-BLED**, Median [IQR] | 2 [1-3] | 1 [1-2] | <0.001 |
| **High Bleeding Risk**, n (%) | 1304 (29.4) | 419 (7.6) | <0.001 |
| *ABC Criteria (n=6091)* |  |  |  |
| **ABC compliant,** n (%) | 560 (23.4) | 1296 (35.1) | <0.001 |
| **Number of ABC criteria fulfilled**, n (%)  0 1 2 3 | 76 (3.2) 558 (23.3) 1204 (50.2) 560 (23.4) | 57 (1.5) 581 (15.7) 1759 (47.6) 1296 (35.1) | <0.001 |
| **‘A’ criterion adherent,** n (%) | 1928 (80.4) | 2696 (73.0) | <0.001 |
| **‘B’ criterion adherent,** n (%) | 1774 (74.0) | 3116 (84.4) | <0.001 |
| **‘C’ criterion adherent,** n (%) | 944 (39.4) | 2175 (58.9) | <0.001 |

Legend: AF: Atrial Fibrillation; BMI: Body Mass Index; CKD: Chronic Kidney Disease; COPD: Chronic Obstructive Pulmonary Disease; DBP: Diastolic Blood Pressure; IQR: Interquartile Range; PAD: Peripheral Artery Disease; SBP: Systolic Blood Pressure; TE: Thromboembolism

**Figure S1 - Kaplan Meier Curves for the risk of MACE according to cluster analysis.**

Legend: p-value for log-rank test


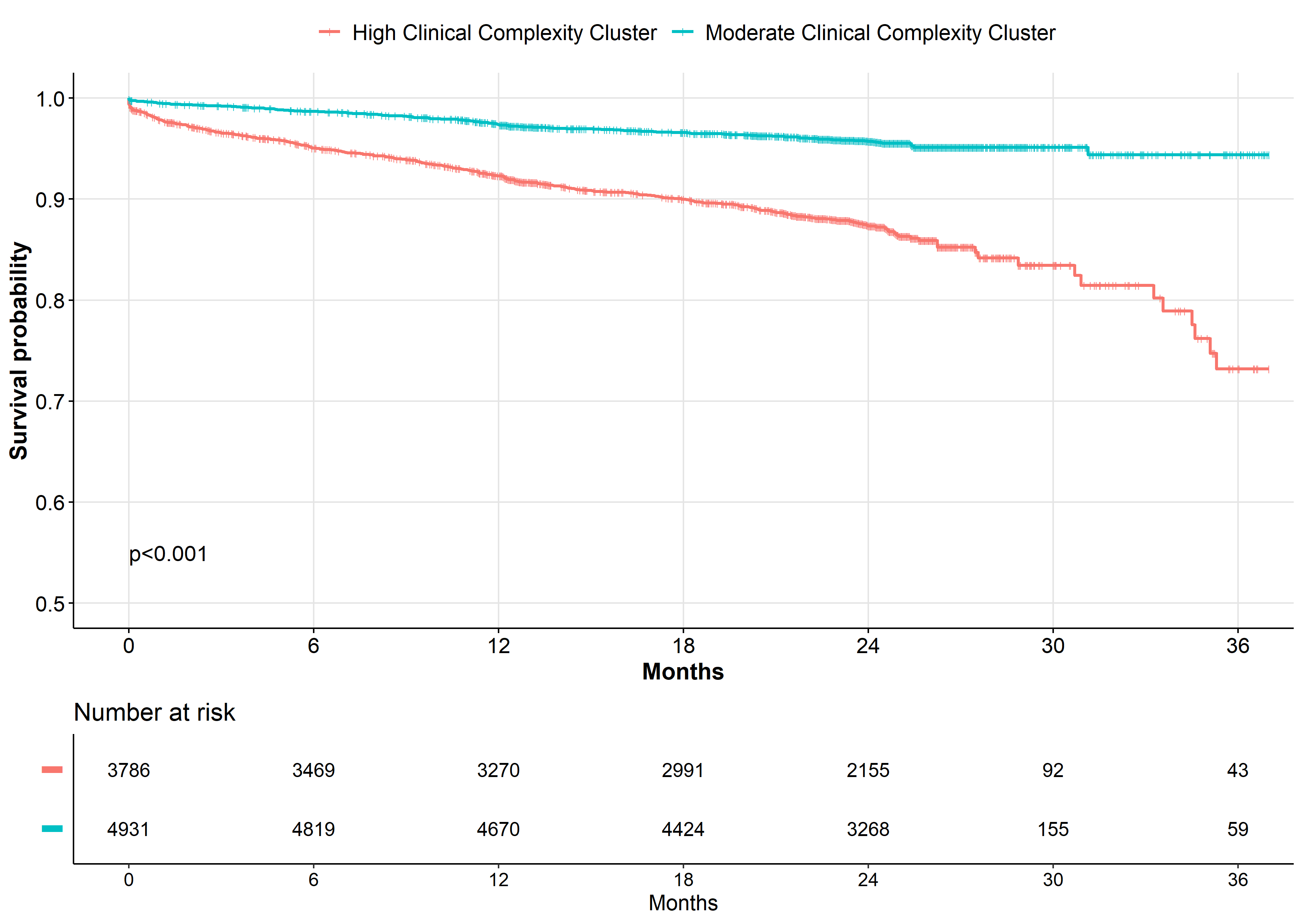


**Figure S2 - Kaplan Meier Curves for the risk of composite outcome according to cluster analysis.**

Legend: p-value for log-rank test


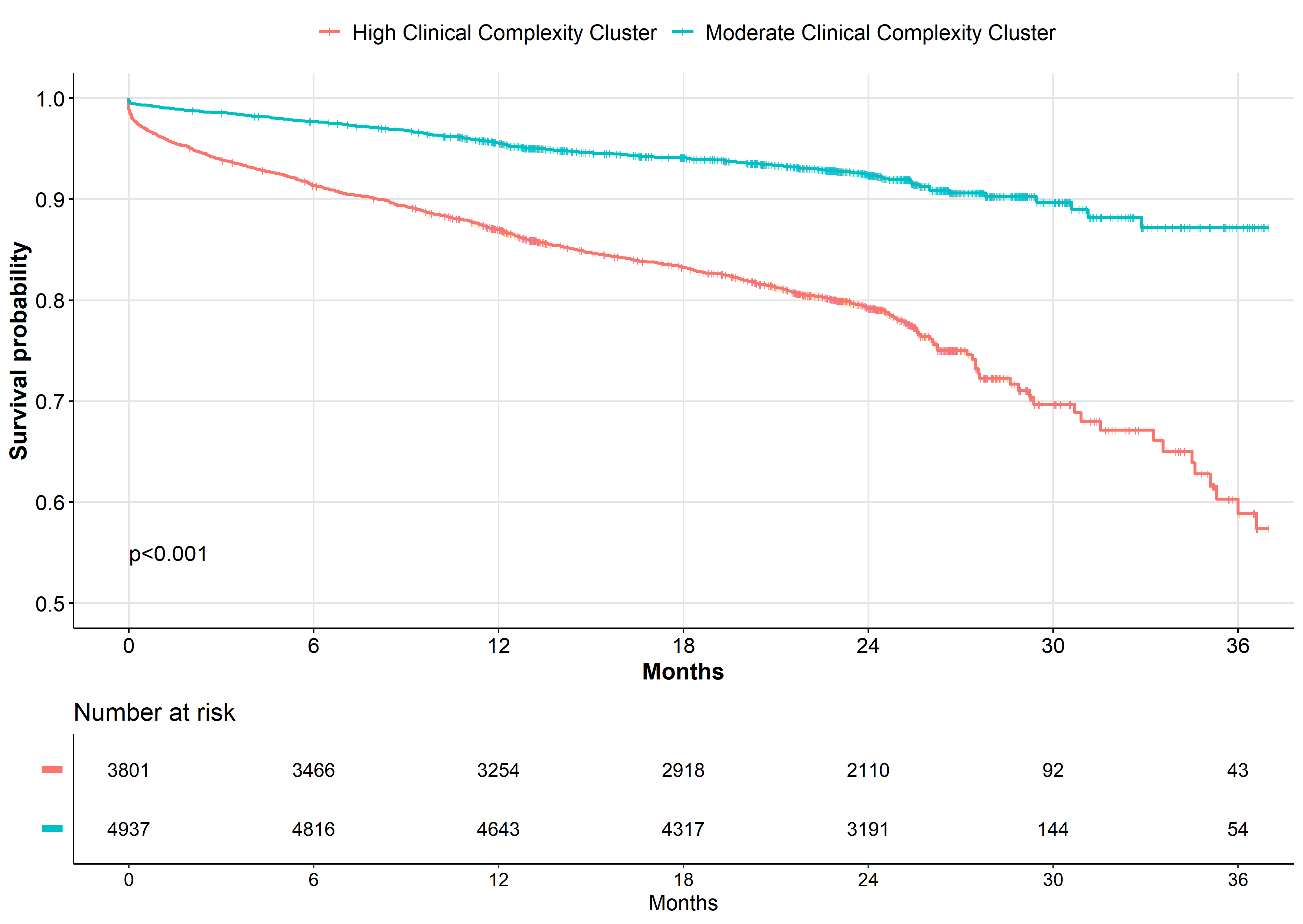


**Figure S3 – Delay of Event analysis for MACE, ABC adherent vs. non-adherent in cluster 1 subgroup**

Legend: DoE=Delay of Event. Figures reported are estimates [95% Confidence Intervals].


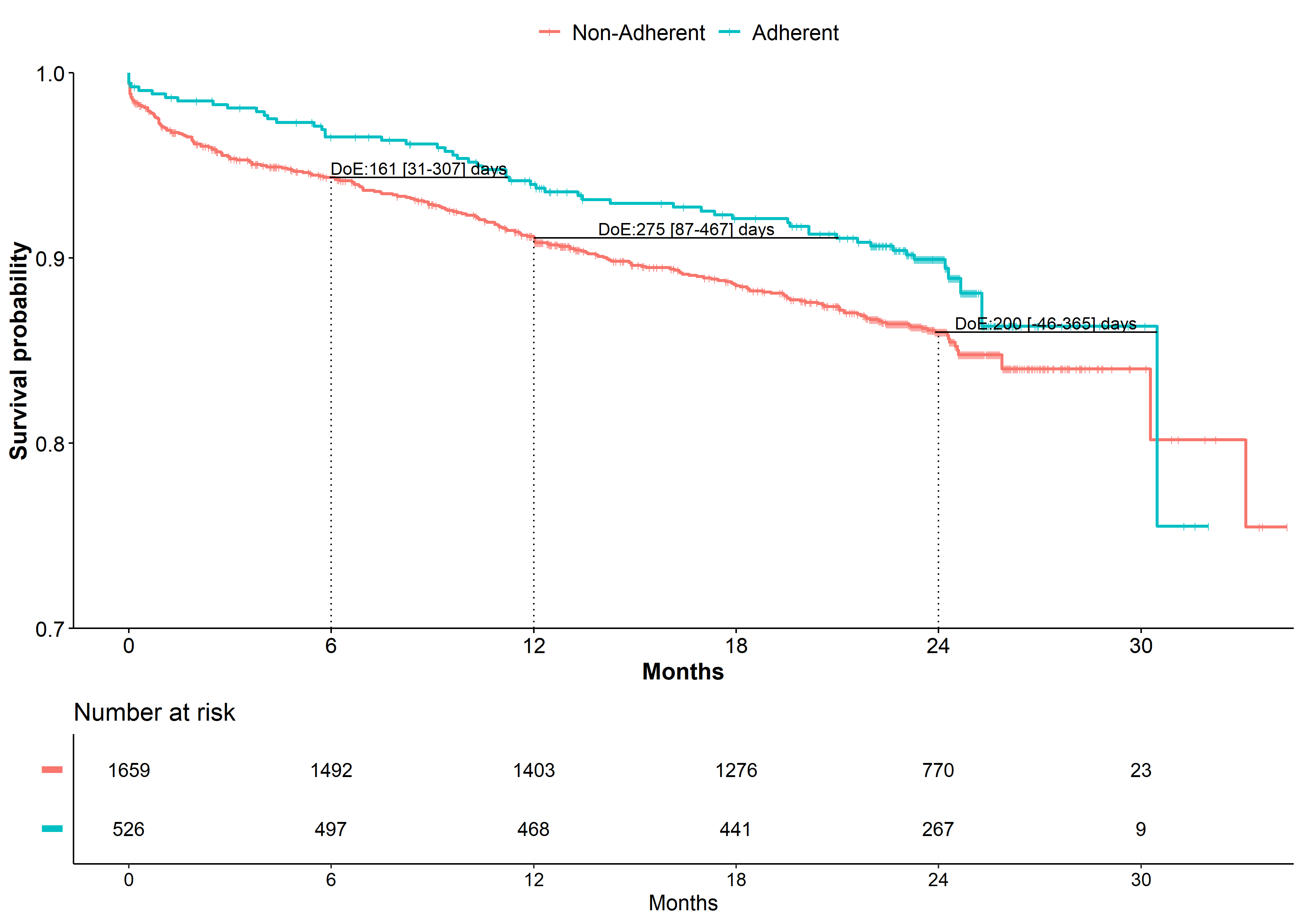


**Figure S4 – Delay of Event analysis for Composite Outcome, ABC adherent vs. non-adherent in cluster 1 subgroup**

Legend: DoE=Delay of Event. Figures reported are estimates [95% Confidence Intervals].


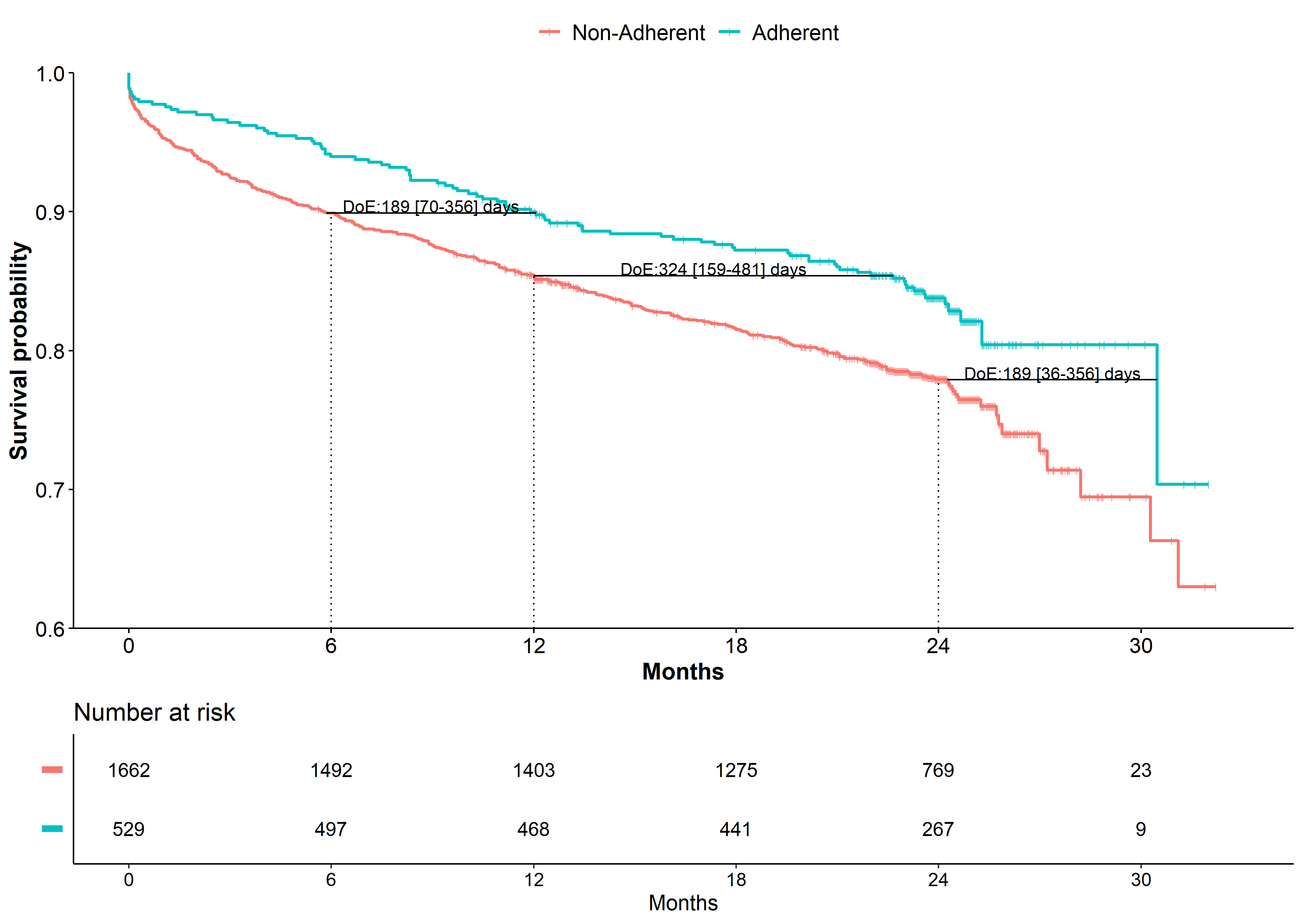

Supplement: Supplementary file 1 — Additional file 1: Table S1. Items Included into the Frailty Index. Table S2. Baseline Characteristics of the Cohort. Table S3. Cox Regression for the risk of major outcomes according to clinical complexity and subgroups. Table S4. Baseline characteristics according to cluster allocation. Figure S1. Kaplan Meier Curves for the risk of MACE according to cluster analysis. Figure S2. Kaplan Meier Curves for the risk of composite outcome according to cluster analysis. Figure S3. Delay of Event analysis for MACE, ABC adherent vs. non-adherent in cluster 1 subgroup. Figure S4. Delay of Event analysis for Composite Outcome, ABC adherent vs. non-adherent in cluster 1 subgroup. [file 12916_2022_2526_MOESM1_ESM.docx]
